# Short Term Depression Unmasks the Ghost Frequency

**DOI:** 10.1371/journal.pone.0050189

**Published:** 2012-12-05

**Authors:** Tjeerd V. olde Scheper, Huibert D. Mansvelder, Arjen van Ooyen

**Affiliations:** 1 Department of Integrative Neurophysiology, Center for Neurogenomics and Cognitive Research, Vrije Universiteit Amsterdam, Amsterdam, The Netherlands; 2 Department of Computing and Communication Technologies, Faculty of Technology, Design and Environment, Oxford Brookes University, Oxford, United Kingdom; Newcastle University, United Kingdom

## Abstract

Short Term Plasticity (STP) has been shown to exist extensively in synapses throughout the brain. Its function is more or less clear in the sense that it alters the probability of synaptic transmission at short time scales. However, it is still unclear what effect STP has on the dynamics of neural networks. We show, using a novel dynamic STP model, that Short Term Depression (STD) can affect the phase of frequency coded input such that small networks can perform temporal signal summation and determination with high accuracy. We show that this property of STD can readily solve the problem of the ghost frequency, the perceived pitch of a harmonic complex in absence of the base frequency. Additionally, we demonstrate that this property can explain dynamics in larger networks. By means of two models, one of chopper neurons in the Ventral Cochlear Nucleus and one of a cortical microcircuit with inhibitory Martinotti neurons, it is shown that the dynamics in these microcircuits can reliably be reproduced using STP. Our model of STP gives important insights into the potential roles of STP in self-regulation of cortical activity and long-range afferent input in neuronal microcircuits.

## Introduction

The phenomenon of the missing fundamental frequency, often referred to as the Ghost Frequency, is the perceived pitch of a harmonic complex when the complex does not contain the pitch frequency itself. The base frequency, which is the pitch that is perceived, is absent from the harmonic complex. The mechanism underlying the perception of the ghost frequency has been the subject of much auditory research and, more recently, model studies. The problem can also be described as determining the lowest frequency that is the multiple of the combined frequencies. A similar phenomenon has been observed in afferent motor input as a ghost motor response [1]. Although it has not yet been shown how sensory systems perceive ghost frequencies, it has been conjectured that noise in sub-threshold activity is a mechanism to improve network performance and robustness [2]. Models that incorporate this type of system noise have shown that, for specific instances, a small network can identify the base frequency of the input [3]. However, the inherent stochasticity does not provide a reliable or highly accurate result across different frequency ranges. The requirement of accurate frequency determination has been argued to be important for pitch perception and subsequent auditory processing [4], [5]. Therefore, the method employed by the auditory system needs to be both accurate and generic in the sense that it does not rely on unique conditions, such as specific network structures or special dynamics. Even though the mechanism for pitch processing has not conclusively been identified, work in the auditory system has demonstrated several properties that indicate contributing components for accurate and efficient frequency coded processing. In primary auditory cortex, it was found that rapid synaptic depression explains non-linear modulation of the spectro-temporal tuning to stimuli [6]. Similarly, variations in conductances allow the processing of acoustic information with high precision in bushy cells cochlear nucleus [7]. Short term suppression and forward-masking in cochlear nucleus [8] and the consistent role of Short Term Facilitation (STF) and Short Term Depression (STD) in the auditory brain stem [9] points to an important function of Short Term Plasticity in highly accurate processing of frequency based sensory input. STP has been widely identified in other areas of the brain as well. Recent work indicates its existence in cortical microcircuits [10], the Calyx of Held [11], thalamo-cortical connections in V1 [12] and numerous other sites. From these experimental results, it can be concluded that STP plays an important role in timing dependent processing throughout the brain in many different areas with different functionality. Synaptic resource models [13], [14] have shown that sensitivity to input frequency and synaptic tuning to synchrony patterns are STP related. We extend this work using a dynamic synaptic activity model to show that Short Term Depression can effectively provide synchrony and frequency summation with high accuracy. With a simple conceptual network, it is shown that the problem of the Ghost Frequency, determining the base frequency of a set of input frequencies, can be resolved using STD. Furthermore, we show, using experimentally derived microcircuits, the likely function of STP within those circuits in self-regulation of cortical activity and long-range afferent input [10].

## Results

### Simple STD Model

**Figure 1:**
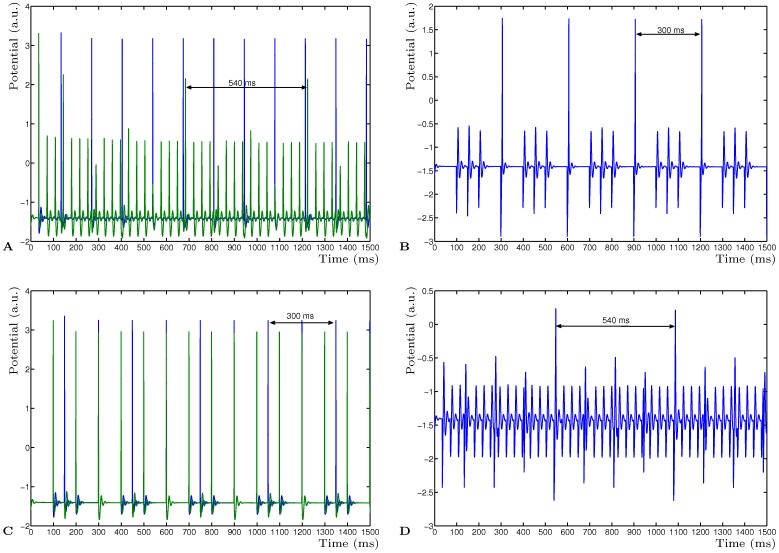
Determining the ghost frequency of two input frequencies. **A** Activity of neurons 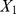 (blue) and 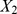 (green) driven by two input frequencies 10 Hz (100 ms) and 6.67 Hz (150 ms). **B** Activity of the read-out neuron 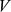 clearly showing the emerging fundamental frequency of 3.33 Hz (300 ms). **C** Neurons 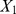 (blue) and 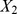 (green) driven by two different input frequencies of 27.78 Hz (36 ms) and 7.4 Hz (135 ms). **D** Activity of neuron 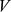 showing the emergent fundamental frequency of 1.85 Hz (540 ms).

A Short Term Depression (STD) synapse model is used to study the pitch perception problem [15] by constructing a mutual inhibitory network with STD (see **Methods** for the description). The frequency encoded input is projected onto two neurons with STD on the mutually inhibiting connections. By comparing the inter-spike interval for both neurons, the emerging periodicity results in the fundamental frequency of the two input frequencies. As a typical example, two mutually inhibitory neurons are driven with input frequencies of 10 Hz (100 ms) and 6.67 Hz (150 ms) (figure 1**A**). These periods are chosen to eliminate any transient effect in the network due to residual activity of the neurons. The auditory system is known to contain very fast neurons, and harmonic sounds seem to be encoded as neural population activity patterns [6], [16]–[19]. Neurons that respond to specific auditory input can repsond with varying discharge rates between approximately 20 to 90 spikes per second [6], [17] with a median at about 70 spikes per second (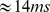.).

The emerging fundamental frequency of 3.33 Hz (300 ms) (figure 1**B**) can be easily read out from the virtual neuron that receives input from all other neurons. Alternatively, providing input to the same two mutually inhibitory neurons with frequencies of 27.78 Hz (36 ms) and 7.4 Hz (135 ms) (figure 1**C**) results in an emerging 1.85 Hz (540 ms) frequency (figure 1**D**). Due to the presence of the STD adaptation, the two activities of the inhibitory neurons synchronise such that each neuron can only become active if it has received inhibitory input from the other neuron, just before receiving input from the input neuron. Without STD, the neurons would simply act as passive coincide detectors and the fundamental frequency would not reliably emerge. The network selects the activity pattern from the combined input activities, which means that any combination of input frequencies will result in an emerging activity which is the Least Common Multiple (LCM) of the input periods. This LCM period forms then the fundamental frequency of those input frequencies. The problem of the ghost frequency can therefore be resolved by simple STD connections of neurons which allows the fundamental frequency of the input to emerge in the network dynamics even when this fundamental frequency is not present in the input. Different frequencies have been used and will consistently result in the emerging LCM for any combination of input frequencies. No combination of input frequencies has been found that failed to result in network dynamics with the LCM, even for very long periods.

**Figure 2:**
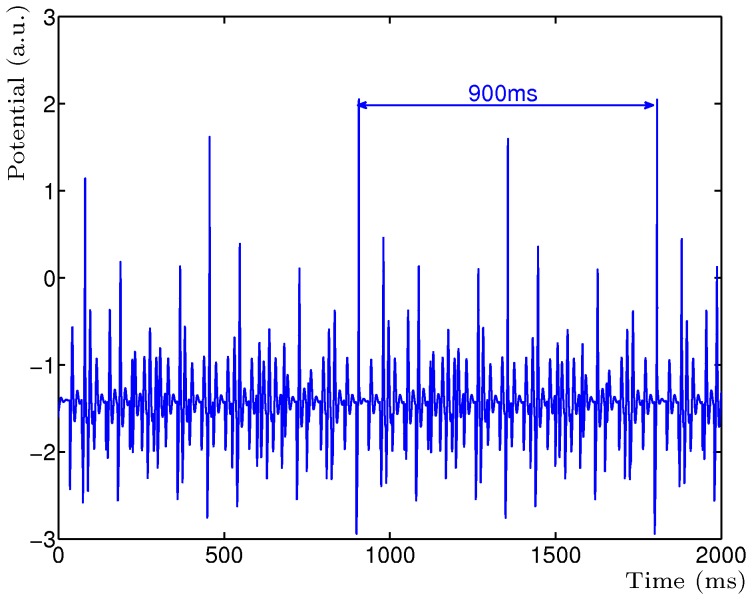
Activity of the read-out neuron 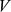 for three input frequencies. Three mutually inhibitory neurons with STD were driven by three frequencies of 13.33 Hz (75 ms), 1.29 Hz (36 ms) and 11.11 Hz (90 ms). The emergent fundamental frequency is 1.11 Hz (900 ms).

The simple STP model can be extended with multiple mutually inhibitory neurons which will allow the network to determine the LCM of multiple input frequencies. For example, presenting three frequencies to a network of three mutually inhibitory neurons with STD, such as 13.33 Hz (75 ms), 1.29 Hz (36 ms) and 11.11 Hz (90 ms), will result in an emerging period of 1.11 Hz (900 ms) (figure 2). Combinations of three frequencies, as well as four or more, will also result in the emerging LCM period of the input frequencies.

**Figure 3:**
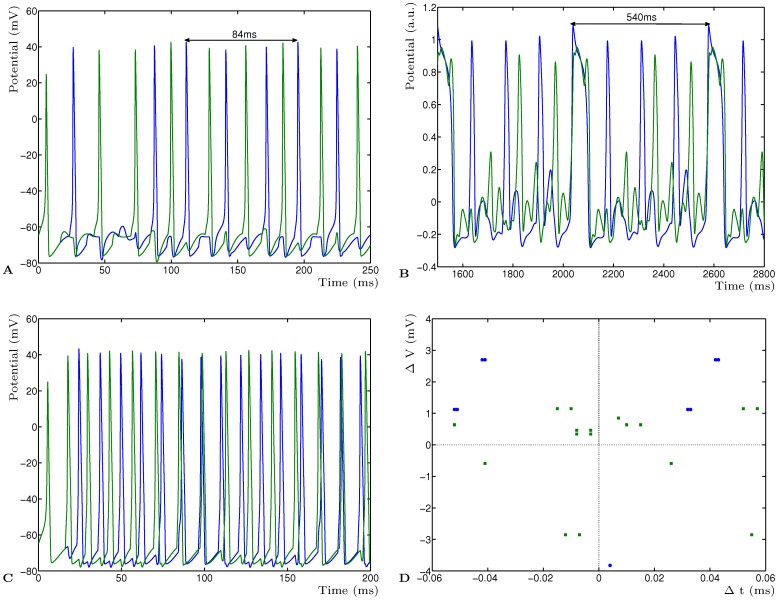
The adaptation of the neural activity by the STD model is independent from the membrane conductance model. **A** Activity of two neurons 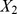 and 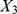 showing the LCM at the fundamental frequency of 11.9 Hz (84 ms) for a network constructed using the Hodgkin-Huxley conductance model. **B** Activity of neurons 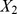 and 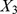 showing the fundamental frequency of 1.85 Hz, using the Fitzhugh-Nagumo model. **C** The network of HH neurons and synaptic transmission as in panel lbfa, but without adaptation. The neurons function as simple coincidence detectors and do not converge to the LCM. Due to the difference in periodicity of the two neurons they will eventually go completely out of phase. **D** Phase diagram of the relative change in membrane voltage for the HH network versus the relative change in timing induced by deterministic LTD adaptation. Shown are the stable phase differences for neurons 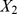 (blue) and 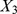 (green) for at least 20 periods. The phase difference was calculated by determining the timing differences of spiking events for individual periods between synapses connecting 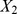 and 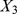 of the adapted network and the non-adapted network. The corresponding difference in amplitude of the membrane activity for each event provides the relative change.

The simple STD model is capable of phase shifting the neural input patterns such that the emergent dynamics becomes the LCM of the input periods. Furthermore, it has three important properties for network dynamics. Firstly, it is model independent in the sense that the model used to simulate the membrane potential does not impact on the functional properties of the STP model. The membrane potential model can be freely exchanged to a different model if desired, provided the input to the STP model is appropriately scaled for the different membrane potential models. The Morris-Lecar model [20] has been used for the simulations described above, and the Hindmarsh-Rose model [21], [22] was used to verify the independence of the STP model. They were found to be equally effective in the emerging LCM network dynamics. We also used the Hodgkin-Huxley model [23] to show the emerging network dynamics for higher frequencies. Input frequencies of 83.33 Hz (12 ms period) and 71.43 Hz (14 ms period) will result in an 11.9 Hz (84 ms) network activity (figure 3 **A**). Even an analytical approximation of the Hodgkin-Huxley model in the form of the Fitzhugh-Nagumo [24] can be used, although the relative stiffness of the two dimensional Fitzhugh-Nagumo model may result in non-physiological spikes (figure 3 **B**). Nevertheless, the STD model still shows the emerging LCM (27.78 Hz and 7.4 Hz resulting in 1.85 Hz, 540 ms period). Lastly, to show that the emerging dynamics is the result of the network architecture in combination with the STD model, the Hodgkin-Huxley simulation described earlier was simulated again but without the synaptic adaptation mechanism (figure 3 **C**). Here simple synaptic transmission was used based on equations (1) and (2) (see Materials and Methods). In this situation the neurons act as simple coincidence detectors in the network and the LCM does not emerge reliably. The LCM would only emerge if the inputs happen to be multiples of each other and the input activities coincide.

**Figure 4:**
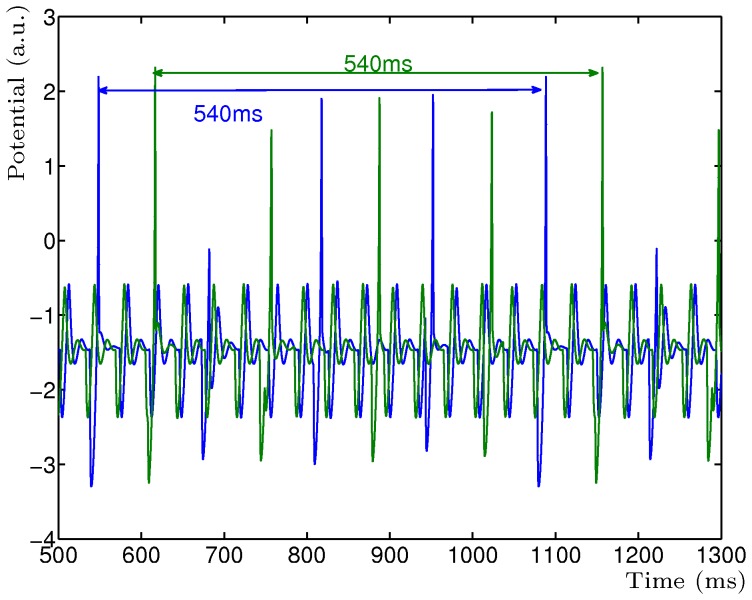
Phase and delay independence of the simple STD model. Two separate simulations of two mutually inhibitory neurons with STD where one simulation has zero phase difference (blue) in input and one simulation has 65 ms phase difference (green) in input. The emergent frequency, as shown by the read-out neuron 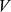, remains the same throughout any phase or delay difference.

The second property is the phase and delay independence of the simple STD model. Because the model does not require specific firing times but combines the input once they occur, given the change in activity pattern, the relative phase of the input does not affect the resulting LCM period of the dynamics. This is illustrated in figure 4 where is shown the LCM of two mutually inhibitory neurons with periodic input of 27.78 Hz (36 ms) and 7.4 Hz (135 ms) (c.f. figure 1**D**). Here, for one simulation, the second periodic input is 65 ms out of phase with the first periodic input. Notice that the entire period is phase-shifted by 65 ms in comparison with the zero phase-shift input simulation but the dynamics is the same. This implies that the model is both phase and delay independent as additional delay in any of the inputs will simply result in a phase-shift in the output but not affect the dynamics in any other way. It is important to recognise that this property is supported by the theory describing Fourier series. It can therefore be conclude that the network performs a correct summation of the input frequencies, resulting in the emerging base frequency. A phase-plot of the Hodgkin-Huxley mutual inhibitory network shows that the phase-shift of the stable oscillating network is consistent and robust (figure 3 **D**). However, the values of the time difference and voltage difference are merely due to the response timings of the conductance model (how quickly the dynamics of the HH model responds to the adaption), it does not say much about the deterministic STP model. Both the exact phase timing and the change in current induced by the STP mechanism are therefore only relevant for the mutual inhibitory network with the chosen neuron models.

**Table 1:** Development of relative error of LCM period for different integrators.

Integrator	RK4	RK-Fehlberg
Stepsize (h)	0.01	0.001
Initial Relative Error ε(*x*_1_)	9.3×10^−5^	2.4×10^−5^
Initial Relative Error ε(*x*_2_)	3.7×10^−5^	9.7×10^−4^
Final Relative Error ε(*x*_1_)	0	0
Final Relative Error ε(*x*_2_)	0	0

The initial period in time has already a small relative error. This error disappears completely over subsequent periods.

The third property concerns the ability of the simple STD model to determine the LCM of the input periods with high accuracy. Different membrane potential models affects the accuracy of the spike timing, some models are more reliable than others due to their intrinsic properties and numerical constraints. Additionally, the method of numerical integration may cause additional errors to emerge. Any computational method that relies on some coincidental occurrence of events will therefore be severely limited if these systematic errors affect the resulting dynamics. The simple STD model compensates for any short term (milliseconds) time differences and is therefore highly accurate. To demonstrate this accuracy, we compared the relative error of the emerging LCM period at the first and last period of the entire simulation of at least 5 LCM periods (Table 1). Due to transients in the dynamics, the first emerging period has a very small relative error, which decreases subsequently and is eliminated at the last (fifth) period. The simulations with Morris-Lecar neurons and STD were integrated with standard Runge-Kutta Order 4 (truncation error 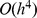) and Runge-Kutta-Fehlberg (truncation error 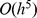).

**Figure 5:**
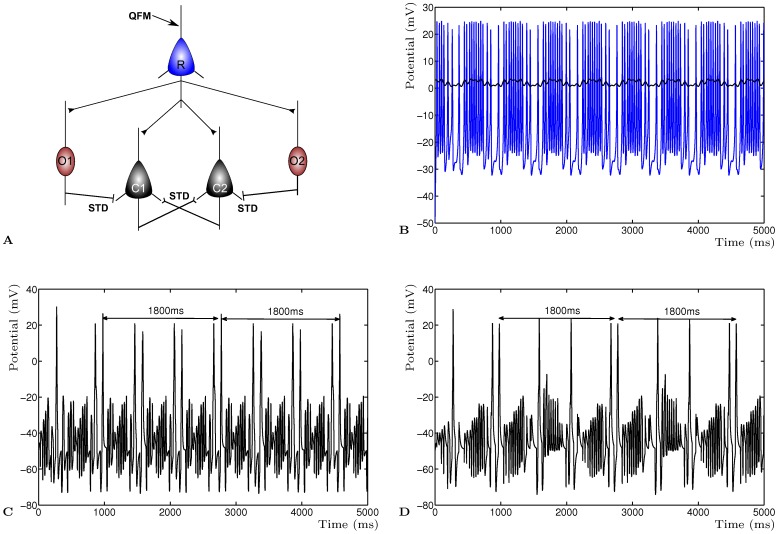
Frequency response model with Short Term Depression. The network neural activity is selected from existing frequencies in the compound input using characteristic activity of the onset neurons. **A** Scheme of a plausible microcircuit in the ventral cochlear nucleus. **B** Burst activity of the receptor neuron 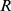 with compound input QFM (black). **C** Chopper neuron 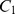 activity resulting in an 1800 ms emergent frequency as part of its unique pattern due to the characteristic frequency of 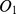. **D** Chopper neuron 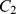 activity with 1800 ms activity pattern and unique activity pattern due to 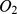.

Note that this accuracy of the activity pattern is visible as accurate replication of repeated inter-spike interval. The actual spiking event timings are dependent on the properties of the membrane potential model and may vary between models and simulation conditions. In auditory nerve representations, it has been found that both rate representation and inter-spike interval representations were accurate for most frequency ranges in response to two concurrent harmonic complex tones with missing fundamentals [25]. This illustrates the high reliability of pitch representations, relatively independent of the coding mechanism which STP can provide.

**Figure 6:**
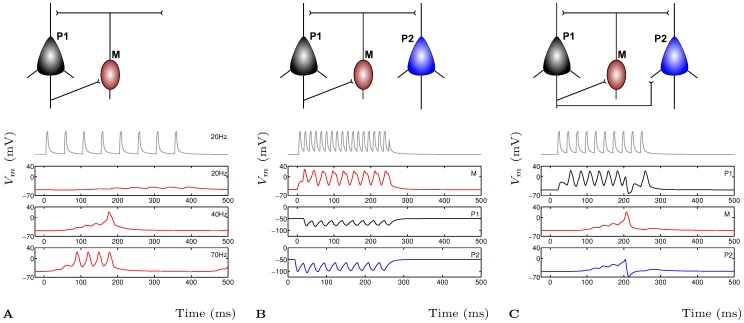
Short Term Plasticity in cortical layer 5 microcircuitry. **A** Short term facilitation of the pyramidal-Martinotti synapse, shown is the activity of the Martinotti cell when the pyramidal cell is stimulated at 20 Hz, 40 Hz and 70 Hz. **B** Two pyramidal and one Martinotti neuron with STF, the Martinotti neuron is stimulated at 70 Hz with adaptive post-synaptic IPSPs at neurons 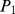 (black) and 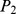 (blue). **C** Complete microcircuit with STF, except the 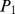 to 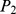 synapse with STD. 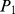 is stimulated at 40 Hz which results in a single AP in 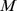 that terminates the stimulation burst in 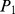 and causes a large IPSP in 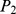.

### Frequency Response Model

As has been shown by the simple STD model, short term plasticity allows the summation of frequencies with high accuracy. An important function of several networks is the extraction of features and frequencies embedded in compound signals. We will describe a plausible biological network, derived from experimental results in the ventral cochlear nucleus with short term plasticity, and determine how STD affects the dynamics of the network.

**Figure 7:**
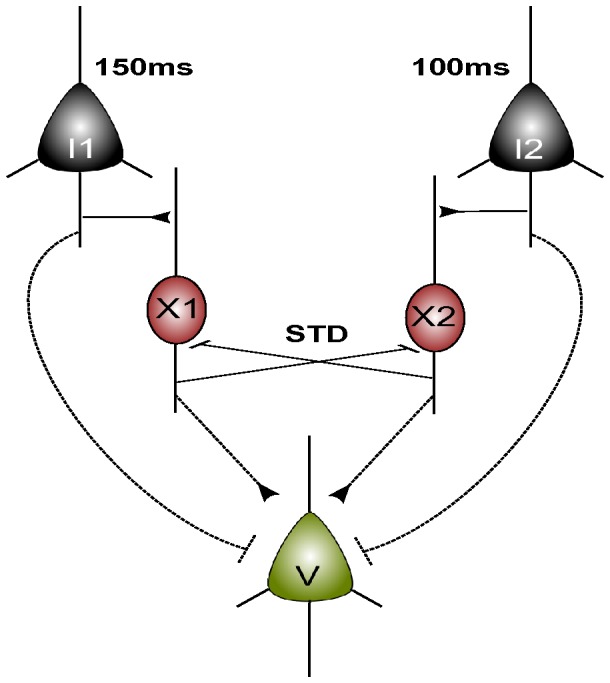
Scheme of the Simple Short Term Depression model. A schematic diagram of two mutually inhibitory neurons with short term depression 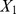 and 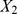. 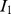 and 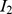 are input neurons which receive periodic input pulses. Neuron 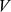 is a virtual neuron which facilitates the readout of neurons 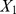 and 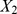 and is not suggested to be biological realistic as such.

In figure 5 **A** is shown the scheme of the biological microcircuit in the ventral cochlear nucleus [26]. Two T-stellate cells (*chopper neurons*) 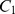 and 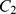 mutually excite each other but are inhibited by D-stellate cells (*onset chopper neurons*) 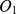 and 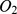 [26]–[28]. Both onset chopper neurons 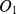 and 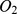 have an internal characteristic frequency which appears as below threshold activity. All neurons receive input from a conceptual receptor cell 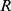 whose activation is determined by the input of an amplitude modulated sine wave in the form of a Quasi Frequency Modulated wave (QFM) (see **Methods**). The excitatory synaptic connections between the chopper neurons and the inhibitory synaptic connections to the chopper neurons are dynamic synapses with Short Term Depression. All neurons receive input from the receptor neuron 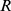 which is based on the QFM signal, in this example 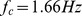 (600 ms period) and 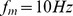 (100 ms period). The receptor neuron responds to the modulated input as a burst pattern correlated with the amplitude modulation of the QFM wave (figure 5 **B**). The onset chopper neurons 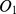 and 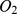 have a characteristic activity of 11.11 Hz (90 ms) and 40 Hz (25 ms). The chopper neurons 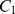 and 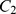 respond to the input from the receptor neuron, one onset neuron and the activity of the other chopper neuron. They show an unique pattern due to the characteristic frequency sensitive inhibitory input from 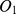 and 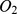, respectively (figure 5 **C** and **D**).

The emerging dynamic activity of the two chopper neurons shows that these neurons select the sub-harmonic activity pattern from the combined activity. They compare the positive input from the other neuron with the frequency sensitive inhibitory input and the receptor input. Such a temporal representation with phase shift has been described in the Ventral Cochlear Nucleus by determining the characteristic behaviour of neurons associated with harmonic sounds [29]. This selection mechanism could function as a selective filter to recode the afferent input into known associated activity patterns. This has important implications for pattern recognition and auditory memory by allowing selected input to be associated with network dynamics.

### Cortical Disynaptic Pathways

Short Term Plasticity is not uniquely a property of the auditory system. In connections between neocortical pyramidal cells STP has been found, as well as in facilitating synapses from mediating inhibitory interneurons. These Martinotti cells can modulate the activity pattern of the pyramidal cells with disynaptic inhibition to regulate cortical activity [10]. The effectiveness of the STP mechanism on this regulation process can be understood by simulating the microcircuit with the appropriate STP properties and showing that the theoretical results converge with the experimental data.

The cortical disynaptic model is based on the micro-cicuit formed by two pyramidal neurons and one inhibitory Martinotti neuron. By changing the connectivity between these three cells, enabling and disabling specific connections, we show how the dynamics of the network is changed due to the STP between connections. Neurons are modelled using Morris-Lecar [20] with STP synaptic connections as described below. Parameter values are used in the same range as for the STD model (see **Methods** for details). In figure 6 is firstly shown a summary of the simulation results which illuminates the function of STP in these types of cortical microcircuits. In panel **A** (top) is shown a minimal network of one pyramidal neuron 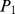 and one Martinotti 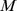 neuron. The pyramidal synapse and the inhibitory Martinotti synapse are short term facilitating which is shown experimentally as evoked APs with higher probability and shorter onset latency. 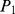 is then stimulated with three trains of neuronal spikes at different frequencies. Shown is the response of the post-synaptic Martinotti cell where, at the lowest frequency of 20 Hz (figure 6 **A** top, with the 20 Hz stimulation pattern), the burst is not strong enough to cause 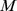 to become active. At 40 Hz (middle) the frequency is just high enough to cause a single action potential at the end of the stimulation burst. At 70 Hz, the Martinotti cell will become active after only two input APs. This sensitivity to frequency and onset latency of the facilitating synapse are well known properties of short term facilitation experimentally [10]. Secondly, in panel **B** of figure 6 (top) is shown a larger cortical microcircuit of two pyramidal neurons 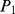 and 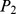 with a single inhibitory Martinotti neuron 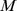. In this situation all synapses are short term facilitating. By stimulating the Martinotti cell 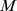 with a single burst of APs at 70 Hz (shown in panel **B** below the network), the two pyramidal neurons show synaptic depression in experimental conditions [10]. Notice that the first two APs cause the second pyramidal neuron 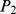 (blue) to be more depressed than the subsequent input APs. This activity dependent synaptic depression is also caused by the facilitating properties of the synapse as has been shown experimentally. Lastly, in panel **C** of figure 6 is shown the full microcircuit which is the same as in panel **B** but with a short term depressing synapse between 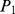 and 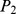. The complete disynaptic connection from 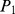 via the Martinotti cell 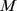 to 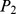 results in a single AP followed by an IPSP in 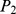 due to stimulation of 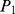 experimentally [10]. 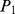 is stimulated with a single burst of APs at 40 Hz (panel **C** below the network), which causes the Martinotti cell 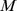 to generate a single post-synaptic AP after several input APs. The second pyramidal cell 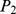 shows EPSPs due to the input from 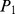 but these are not sufficient to generate an AP. When 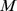 becomes active, 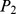 shows a strong IPSP due to the input from 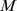. The activity from 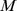 will also cut short the burst from 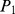 which then regenerates the burst just before stimulation ends.

The simulation results match excellently experimental results describing both STD and STP in this microcircuit [10] which validates both the experimental results and the STP model as realistic representations of the mechanism of STP in cortical microcircuits. Recent work on the intra-striatal microcircuit has shown similar results for different dynamic interactions due to plasticity modulations of different pathways [30].

## Discussion

In pitch theory, different schemes for pitch estimation are used, based on either pattern matching or autocorrelation. Both are used to perform the basic operation of period estimation needed to identify the pitch [31]. Short Term Plasticity can provide an answer to this aspect as a mixture of the two methods. It selects appropriate periods from frequency coded input (or characteristic frequencies) as well as identifying the presence of relevant periods in the network activity. This is particularly useful for high fidelity information processing at high frequency to enable fine tuning to which STP may well be suited [32]. Further properties of the dynamic STP network, such as linear summation, contributes to the determination of masking and efficient encoding within the auditory system [18], [33], [34].

Sounds other than harmonic complexes may be perceived as pitch, such as periodic and aperiodic click trains [5]. The STP model would hold for these types of sounds because the perceived pitch as the LCM of the combined inputs due to STD would still emerge. In particular, emerging global pitch of an aperiodic input in the presence of background noise would be conceivable as a matched periodicity of temporally limited input within the random background. Aspects such as the increased accuracy of pitch discrimination over stimulus duration [5] can also be recognised within the STD model, where events are matched as they occur and improve their accuracy during a longer stimulus. Also, the ability to match a pitch over noise filled gaps is conceivable, as long as the matching events occur sufficiently often to maintain the temporal LCM of the pitch. Noise would not disrupt the perception as it would not cause a network response. Specific constraints of the STP model for complex pitch simulations remain to be defined and may form the basis of some more interesting work.

The deterministic nature of the STP model makes it eminently useful to study the basic computation that the STP mechanism allows a mico-circuit to perform. That micro-circuits are capable of such computation has long been suspected but never really shown computationally. It is not possible to reproduce our results with the STP model by Tsodyks et al. [13], [14] as that model does not include a mechanism for time shifts. The explicit time variable in that model is not amenable to adjustment. The stochastic model of perceived pitch [3] suffers from a limitation based on the nature of randomness. The probability of a matching LCM period can also be cancelled out with the probability of a non-matching period, which is why the stochastic model occasionally misses a period. It is also inherently inaccurate due to the presence of the stochastic adaptation.

The consequences of the dynamic STP model go beyond the auditory system. As has been shown by the frequency response model of the chopper neurons, the excitatory and feed-forward inhibitory synapses work synergistically as adaptive filters of spike trains which has been shown for hippocampal synapses as well [35]. This aspect takes STP to a more central role in accurate information processing of complex neural spike patterns [36]. Increased accuracy, phase shifting, enhanced phase locking to input, and selecting characteristic features from the spike pattern are cardinal elements of the function of dynamic short term plasticity within the synapse and neural networks.

## Methods

### Dynamic Short Term Plasticity Model

The aim of the dynamic short term plasticity model is to define a simple representation of synaptic plasticity that allows temporal adjustments. This is achieved by using a dynamic systems approach instead of a time constants methodology such as would be the case with an alpha function [37]. In a manner similar to the definition of an alpha function [37], the post-synaptic activity can be approximated by two simple rate equations as follows.
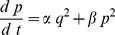
(1)

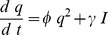
(2)where 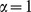 or 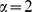, 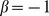, 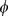 is in the range 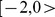, 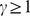, 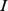 is the external input, 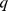 the input variable and 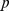 represents the synaptic activity. The synaptic strenght is simply represented by the scalar 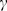 and is adjusted for appropriate input to the chosen neural model. Short Term Plasticity can subsequently be implemented using a resource based model suggested by Tsodyks et al [13], [14]. It is added to equations (1) and (2) by an additional variable 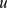 as below.
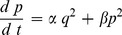
(3)

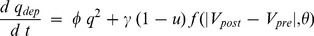
(4)

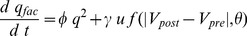
(5)

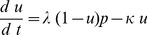
(6)where 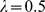 and 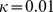. The function 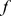 represents the relative activities of the pre- and postsynaptic cell with threshold 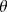. 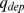 (4) is used to implement Short Term Depression and 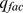 (5) is used to implement Short Term Facilitation, and are not used concurrently. The membrane potential terms are based on standard models such as the Morris-Lecar [20], the Hindmarsh-Rose model [21], the Fitzhugh-Nagumo [24] or the standard Hodgkin-Huxley model [23]. A network consists of membrane potential models in one or more compartments with additional synaptic connections of the form of (3), (4) and (6) for STD and (3), (5) and (6) for STF.

### Simple Short Term Depression Model

To study the effect of Short Term Depression, a simple network of two mutually inhibitory neurons was constructed with two input neurons and a read-out neuron. In figure 7 is shown the microcircuit with the five model neurons. Input neurons 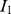 and 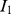 receive periodic input pulses of fixed frequencies. These pulses are presented to the input neurons as synaptic inputs using equations (1) and (2) with appropriate synaptic scaling to ensure a post-synaptic response in the input neurons. The synaptic inputs are formed by a square input pulse with a period equal to the chosen drive period. The two input neurons project onto inhibitory neurons 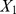 and 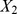 which inhibit each other with short term depression. Neuron 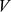 is a virtual neuron that compares the behaviour of 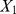 and 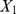 with the input from 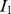 and 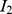. Neuron 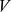 could be replaced by a more realistic network, such that additional interneurons would provide the input, but this would make the network needlessly complex. The model concerns the effect of STD on the two mutually inhibitory neurons, and neuron 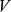 simply facilitates the read-out of the emergent dynamics of neurons 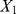 and 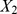. Parameter values are 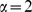, 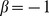, 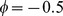, and 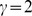.

### Quasi Frequency Modulated Wave

To provide a realistic frequency encoded compound wave to the model input neurons of the Frequency response model, a Quasi Frequency Modulated wave (QFM) was constructed [27]. This compound wave of one carrier frequency and one modulating frequency has sufficient depth and complexity to form an input neuron bursting pattern.
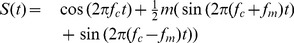
(7)where 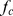 is the carrier frequency, 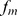 modulation frequency and 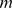 the modulation depth. The receptor neuron in figure 5 **A** receives this wave as input, this results in a neural activity pattern of a time modulated burst as shown in figure 5 **B**.
